# Peptide‐PAINT Enables Investigation of Endogenous Talin with Molecular Scale Resolution in Cells and Tissues

**DOI:** 10.1002/cbic.202100301

**Published:** 2021-07-30

**Authors:** Lisa S. Fischer, Thomas Schlichthaerle, Anna Chrostek‐Grashoff, Carsten Grashoff

**Affiliations:** ^1^ Department of Quantitative Cell Biology Institute of Molecular Cell Biology University of Münster Schlossplatz 5 Münster 48149 Germany; ^2^ Department of Biochemistry University of Washington Seattle WA 98195 USA; ^3^ Institute for Protein Design University of Washington Seattle WA 98195 USA

**Keywords:** cell adhesion, peptides, protein-protein interactions, single-molecule studies, talin

## Abstract

Talin is a cell adhesion molecule that is indispensable for the development and function of multicellular organisms. Despite its central role for many cell biological processes, suitable methods to investigate the nanoscale organization of talin in its native environment are missing. Here, we overcome this limitation by combining single‐molecule resolved PAINT (points accumulation in nanoscale topography) imaging with the IRIS (image reconstruction by integrating exchangeable single‐molecule localization) approach, enabling the quantitative analysis of genetically unmodified talin molecules in cells. We demonstrate that a previously reported peptide can be utilized to specifically label the two major talin isoforms expressed in mammalian tissues with a localization precision of <10 nm. Our experiments show that the methodology performs equally well as state‐of‐the‐art single‐molecule localization techniques, and the first applications reveal a thus far undescribed cell adhesion structure in differentiating stem cells. Furthermore, we demonstrate the applicability of this peptide‐PAINT technique to mouse tissues paving the way to single‐protein imaging of endogenous talin proteins under physiologically relevant conditions.

## Graphical abstract

**The PIPKIγ‐peptide‐PAINT** approach is introduced in this study to enable quantitative investigation of the cell adhesion protein talin with single‐molecule resolution in cells. The technique does not require genetic modifications and allows the analysis of endogenously expressed molecules. The first application reveals atypical adhesion structures in differentiating mesenchymal stem cells.

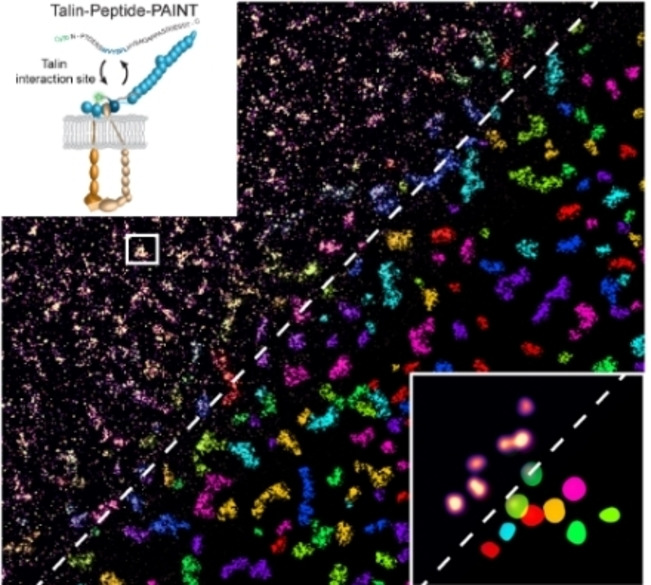

## Introduction

The adhesion of cells to their extracellular environment is key for the development of multicellular organisms and central to a wide range of cell biological processes.^[1]^ Cell‐matrix adhesion is mediated by integrin receptors that specifically bind extracellular ligands and connect through adaptor proteins to the intracellular actin cytoskeleton.^[2]^ The protein that is central for the regulation of this process and thus key to cell‐matrix adhesion is called talin.^[3]^ Talin mediates its function by directly binding and thereby activating integrin receptors with an N‐terminal FERM domain;^[4]^ at the same time, talin connects with its C‐terminal rod domain to the actin cytoskeleton and thereby establishes mechanosensitivity during cell adhesion.[^5^, ^6^] Furthermore, talin recruits a number of molecules such as vinculin to the adhesion structure and thereby promotes the formation of a multimolecular complex called focal adhesion (FA).[^1^, ^7^] It has been recognized that each of these talin functions are indispensable for proper cell attachment[^5^, ^8^] and since talin is expressed in all cell types, it is crucial for the development and homeostasis of many tissues^[9]^ and relevant to a range of pathologies including immunological disorders^[10]^ and cardiovascular disease.^[11]^

Given its important function, it is not surprising that talin is a thoroughly studied cell adhesion molecule, and many of its biochemical and biophysical properties have been unraveled.[^3^, ^12^] Yet, how talin functions on molecular scales within FAs is still poorly understood because suitable techniques to quantify talin at single‐molecule resolution in cells were missing. This started to change with the development of super‐resolution microscopy approaches^[13]^ – and especially those techniques that permit the visualization of individual proteins in cells. Interferometric photoactivated localization microscopy (iPALM) was utilized to resolve, albeit not yet with molecular resolution, the horizontal organization of prominent FA proteins confirming the central role of talin that spans the entire adhesion structure by connecting integrins with the actin cytoskeleton.^[14]^ Single‐protein tracking photoactivation localization microscopy (sptPALM) demonstrated that talin immobilizes integrin receptors in FAs consistent with its function as a mechanical linker,^[15]^ and our own DNA‐points accumulation in nanoscale topography (PAINT)‐based experiments showed how talin molecules assemble during cell attachment. These experiments also revealed the formation of a ternary complex between integrin β1, talin‐1, and a second integrin activator called kindlin‐2.^[16]^ Overall, the studies emphasize that fully understanding talin function requires techniques enabling quantitative, molecular‐scale analyses in cells.

**Figure 1:**
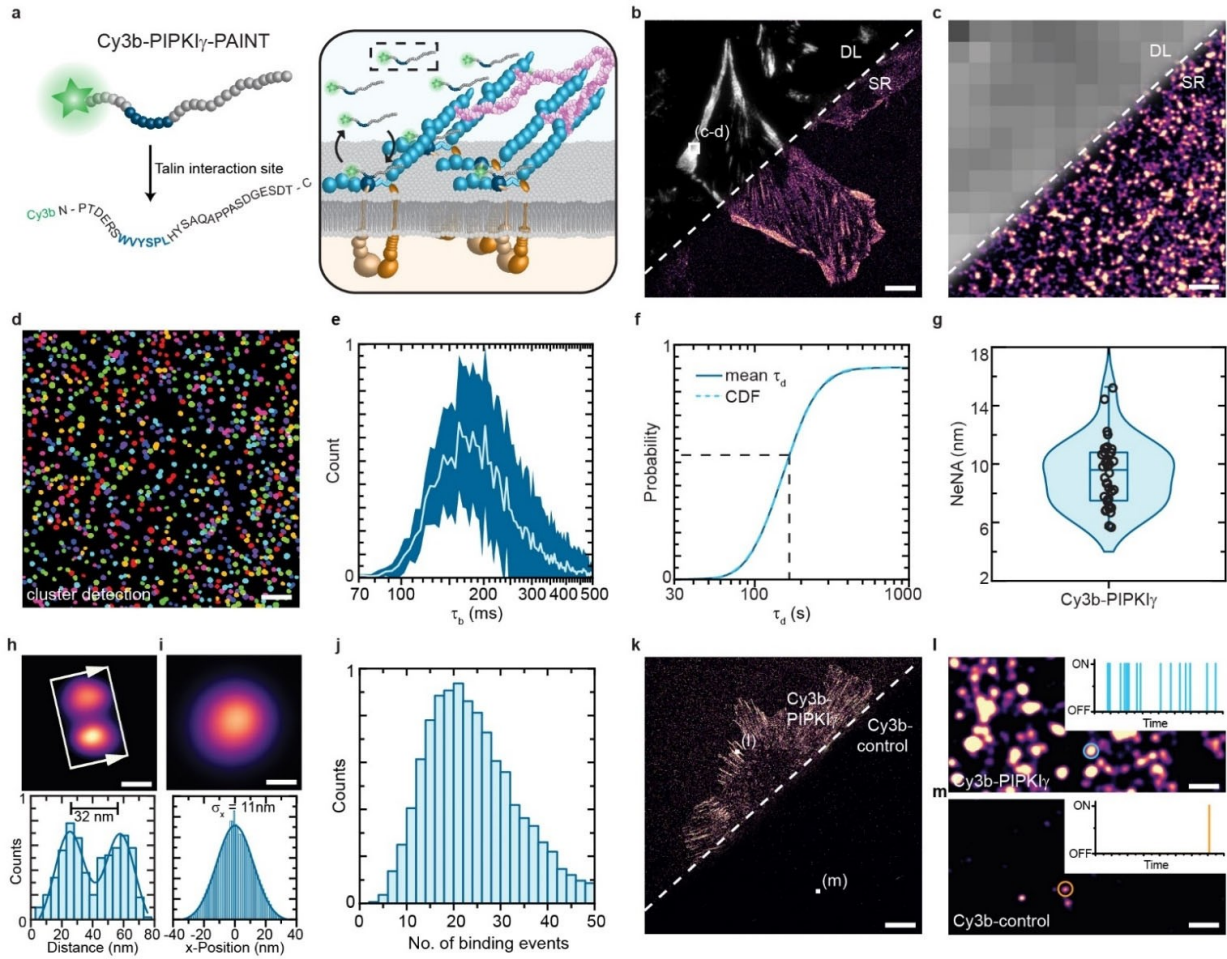
PIPKIγ‐PAINT allows single‐molecule resolved measurements in cells. **a**) Schematic overview of the PIPKIγ‐PAINT concept. The strategy uses a previously described,[^19^, ^20^] 28 amino acid (aa) long peptide (PIPKIγ) with a Cy3b modification at the N‐terminus (Cy3b‐PIPKIγ). The peptide binds transiently through a distinct interaction motif (blue) to the FERM domain of talin. **b**) Side‐by‐side view of a diffraction‐limited (DL) image of paxillin localization in focal adhesions (FAs) and super‐resolved (SR) Cy3b‐PIPKIγ‐PAINT image of endogenous talin. **c**) Zoom‐in of the outlined area in **b** shows the diffraction limited paxillin image and a super‐resolved view of talin localization clouds in FAs. **d**) Single‐molecule detection of talin using an automated cluster detection algorithm based on a modified Ripley's K function. **e**) Distribution of the ON time (τ_b_) for Cy3b‐PIPKIγ (mean τ_b_=223 ms; n=10,843 localization clouds). **f**) The mean dark time of Cy3b‐PIPKIγ was determined by fitting the cumulative distribution function of the dark times (n=10,843 localization clouds). **g**) Evaluation of the localization precision by NeNA‐based analysis of all cells imaged with Cy3b‐PIPKIγ indicates an average localization precision of 9.6 nm (n=40 cells). **h**) Separation of distinct talin localization clouds and cross‐sectional histogram analysis reveals high‐resolution PAINT imaging using Cy3b‐PIPKIγ. Arrows indicate the plotting direction of the histogram, with arrowheads pointing towards the x‐axis. **i**) Average of 10,843 single talin localization clouds obtained by PIPKIγ‐PAINT aligned to their center‐of‐mass. Gaussian fit of aligned 10,843 single talin localization clouds indicates a localization precision of 11 nm. **j**) Quantitative PAINT evaluation of individual talin localization clouds reveals unimodal distribution of binding events (mean=24), indicating molecular resolution imaging (n=10,843 localization clouds). **k**) Side‐by‐side view of Exchange‐PAINT images acquired with Cy3b‐PIPKIγ and the scrambled control peptide (Cy3b‐control). **l**) Zoom‐in of outlined area in **k** shows repetitive binding events indicative of a specific interaction. **m**) Zoom‐in of outlined area in **k** reveals isolated, non‐repetitive binding events. Boxplots show median and 25^th^ and 75^th^ percentage with whiskers reaching to the last data point within 1.5×interquartile range. ON time distributions show mean±standard deviations. Scale bars: 6.5 μm (**b**), 3.5 μm (**k**), 200 nm (**c**, **d**), 130 nm (**l**, **m**) 40 nm (**h**) 20 nm (**i**).

An important limitation of the currently available single‐molecule localization technologies is that they do not enable the analysis of talin in its native state. Experiments typically use a genetically modified talin (fused with a fluorophore or labeling‐tag), which is then re‐expressed in cells and labeled (or photoactivated) to perform super‐resolution microscopy.[^14^, ^15^, ^16^] Since certain cell lines and tissue samples are inaccessible to genetic modification, however, important aspects of talin biology (and thus cell adhesion) remain unexplored on the nanoscale. For instance, how talin proteins assemble in 3D environments or even tissues ‐ and how, in general, FAs are structured in vivo – is largely unknown.[^17^, ^18^] Furthermore, there is reasonable concern that non‐natural expression systems are limited in capturing the complex biology of the endogenously expressed proteins; effects of distinct splice isoforms, for instance, cannot be easily explored when only one particular cDNA construct is expressed in cells. To overcome these limitations, we here combine the power of PAINT imaging,^[21]^ which allows single‐protein resolved analyses in cells, with the IRIS approach in which protein fragments are used to transiently label endogenous molecules for super‐resolution analysis (Figure 1a).^[19]^ Our experiments demonstrate that a previously identified peptide recognizes both major talin isoforms expressed in mammals, talin‐1 and talin‐2, and can be used in a Peptide‐PAINT approach to enable the nanoscale analysis of talin in unmodified cells. In contrast to a previously reported Peptide‐PAINT methodology,^[22]^ however, the technique directly labels the target protein and is also applicable to tissue sections allowing the investigation of talin in its native environment.

Thus, the here developed technology should be highly useful to study talin – and in extension cell‐matrix adhesions – with molecular‐scale resolution in a broad range of contexts.

## Results and Discussion

### The PIPKIγ peptide allows single‐protein resolved talin imaging in cells using PAINT

The here described strategy is based on a previously published probe, which was implemented in the IRIS (image reconstruction by integrating exchangeable single‐molecule localization) approach, where small protein fragments are utilized to specifically but transiently label endogenous molecules for super‐resolution imaging.^[19]^ The previous IRIS study labeled FAs with a 28 amino acid (aa) long peptide, derived from phosphatidylinositol (4) phosphate 5 kinase type Iγ (PIPKIγ), but did not specify which FA resident proteins are recognized. Since PIPKIγ directly interacts with talin,[^20^, ^23^] we wanted to explore whether the PIPKIγ peptide could be employed to specifically study talin localization in cells (Figure 1a). We also wondered whether the peptide labeling approach, which was previously utilized with single‐molecule speckle microscopy,[^19^, ^24^] could be combined with PAINT microscopy^[21]^ to allow quantitative, single‐molecule resolved imaging. PAINT is a super‐resolution technique that benefits from a comparably simple microscopy setup and is increasingly used as DNA‐PAINT to study molecules with single‐digit, nm‐scale resolution.^[25]^ In addition, PAINT enables the precise quantification of the visualized molecules, for example through a procedure called quantitative PAINT (qPAINT),^[26]^ and allows multiplexed experiments by Exchange‐PAINT.^[27]^

To systematically test these ideas, we used a Cy3b‐conjugated PIPKIγ‐peptide to label immortalized mouse embryonic fibroblasts (MEFs) that were adherent to glass coverslips and thus expected to form FAs. Consistent with the previous study,^[19]^ Cy3b‐PIPKIγ marked FAs in our cells as indicated by the presence of the FA marker paxillin (Figure 1b, c). To allow PAINT analyses, we repeated the experiment by incubating fixed cells with Cy3b‐PIPKIγ and imaging them with a total internal reflection fluorescence (TIRF) microscope. Indeed, the data revealed FA‐specific, repetitive ‘blinking’ events that are typically used in super‐resolution approaches to reconstruct images with sub‐diffraction resolution (Supporting Video 1). To analyze these data in an automated fashion, we updated our previously developed data analysis pipeline,^[16]^ which uses consecutive filtering steps to distinguish specific from non‐specific signals, with a Ripley's K function‐based cluster detection algorithm^[28]^ to improve signal segmentation. Evaluation of the Cy3b‐PIPKIγ labeled cells using this approach revealed the presence of distinct talin localization clouds (Figure 1c, d) reminiscent of the previously observed DNA‐PAINT based talin signals obtained in genetically modified cells.^[16]^ At the here used peptide concentration (7 nM), we observed an average bright time (τ_b_) of around 223 ms±98 ms and a mean dark time (τ_d_) of 168 s±75 s (Figure 1e, f; Supporting Table S2), similar to the observed ON‐ and OFF switching of DNA imager strands used in DNA‐PAINT‐based approaches.^[25]^

The overall localization precision of all acquired images was determined to be 9 nm using nearest neighbor analysis (NeNA),^[29]^ and the analysis of 10.843 individual localization clouds, aligned to their center of mass, indicated an average localization precision of about 11 nm (Figure 1g–i; Supporting Figure S1). The symmetric and homogenous signal distribution isolated from individual localization clusters indicated that the data originated from the interaction of Cy3b‐PIPKIγ with a single interaction site, and plotting the average number of binding events for all 10.843 molecules during the time of acquisition resulted in a unimodal distribution (Figure 1j). As the number of binding events per localization cloud directly correlates with the number of docking sites on the target molecule,^[26]^ these data suggest the detection of single talin proteins using Cy3b‐PIPKIγ.

To confirm the specificity of the Cy3b‐PIPKIγ signal, we used a Cy3b‐conjugated scrambled version of the peptide (Cy3b‐control; Supporting Table S1) and followed the same labeling protocol as before. Incubation with the Cy3b‐control peptide led to isolated localization clouds only, which were characterized by non‐repetitive binding events typically observed for non‐specific interactions (Figure 1k‐m; Supporting Figure S2). To provide direct evidence for talin specificity, we used Cy3b‐PIPKIγ to image mouse kidney fibroblasts (MKFs) genetically depleted of talin‐1 and talin‐2 (*Tln1* ^−/−^ *Tln2* ^−/−^)[^5^, ^30^] but did not observe any specific signals in these cells (Supporting Figure S3). Together, these experiments demonstrate that the combination of the PIPKIγ peptide with PAINT imaging allows the specific, single‐protein resolved analysis of individual talin molecules in unmodified cells.

### Specific detection of talin‐1 and talin‐2 using PIPKIγ‐PAINT

**Figure 2:**
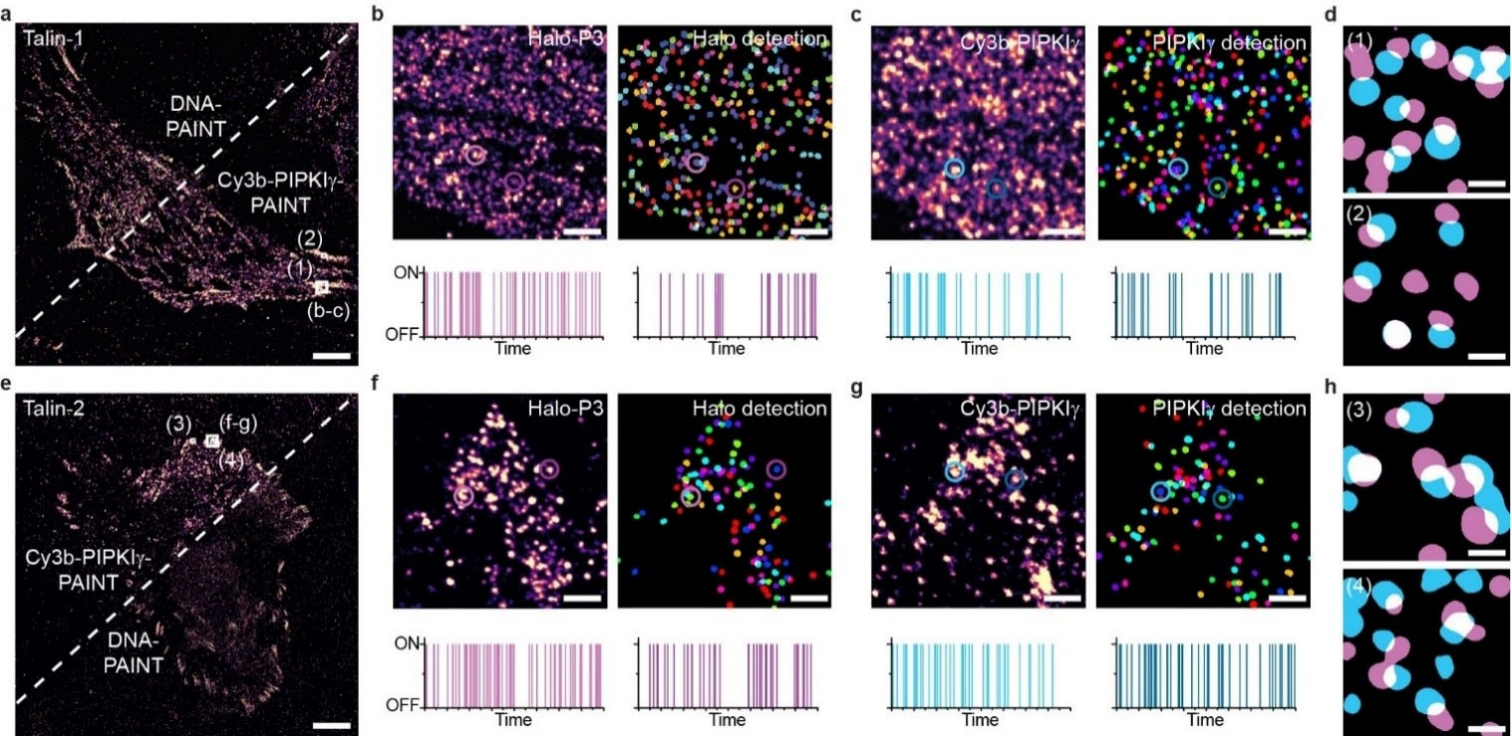
PIPKIγ‐PAINT allows the detection of both major talin isoforms. **a**) Overlay of Exchange‐PAINT data from DNA‐PAINT and PIPKIγ‐PAINT of a talin‐1‐HaloTag expressing cell (Talin‐1). **b**) Zoom into the focal adhesion (FA) area outlined in **a** shows single‐labeled talin localization clouds acquired with a P3 imager strand and the corresponding image upon automated localization cloud detection. **c**) Zoom into FA area outlined in **a** shows single‐labeled talin localization clouds acquired with Cy3b‐PIPKIγ and the localization cloud detection using an automated analysis pipeline. **d**) Zoom‐ins highlighted in **a** reveal co‐localization of Cy3b‐PIPKIγ (blue) and DNA‐PAINT (magenta) signals both labeling talin‐1. **e**) Exchange‐PAINT experiment with a talin‐2‐Halo expressing cell (Talin‐2). Overlay displays a cell consecutively imaged with PIPKIγ‐PAINT and DNA‐PAINT. **f**) Zoom into FA area of a talin‐2 expressing cell outlined in **e** shows single‐labeled talin‐2 localization clouds acquired with P3 imager strand and detection of talin‐2‐HaloTag localization clouds. **g**) Zoom into FA area outlined in **e** shows single talin‐2 localization clouds acquired with Cy3b‐PIPKIγ in talin‐2 expressing cells and the corresponding cluster detection image. **h**) Zoom‐ins highlighted in **e** reveal co‐localization of Cy3b‐PIPKIγ (blue) and DNA‐PAINT (magenta) signals of talin‐2. Scale bars: 5 μm (**a**, **e**), 220 nm (**b**, **c**, **f**, **g**), 40 nm (**d**, **h**).

Mammalian cells express two talin proteins,^[31]^ talin‐1 and talin‐2, and many tissues such as heart muscle or kidney co‐express both variants.[^32^, ^33^] We therefore wanted to test whether both talin isoforms can be detected with the PIPKIγ‐PAINT approach and used *Tln1* ^−/−^ *Tln2* ^−/−^ MKFs that re‐expressed either talin‐1 or talin‐2 constructs. To control these experiments, we fused both isoforms with a HaloTag that can be labeled with a chloroalkane‐modified DNA docking strand. We then performed DNA‐PAINT based measurements using the labeled HaloTag as a target for the complementary imager strand (P3 imager strand, Supporting Table S3),^[16]^ which revealed the expected localization signal of talin‐1 and talin‐2 in FAs. Next, we performed Exchange‐PAINT to image the same cells using Cy3b‐PIPKIγ. Indeed, PIPKIγ‐PAINT imaging resulted in a very similar localization pattern when compared with the DNA‐PAINT measurements and highly repetitive signals indicating specific interactions (Figure 2a–h). Thus, Cy3b‐PIPKIγ is capable of efficiently detecting both talin isoforms.

### PIPKIγ‐PAINT enables high quality, single‐protein resolved quantifications

**Figure 3:**
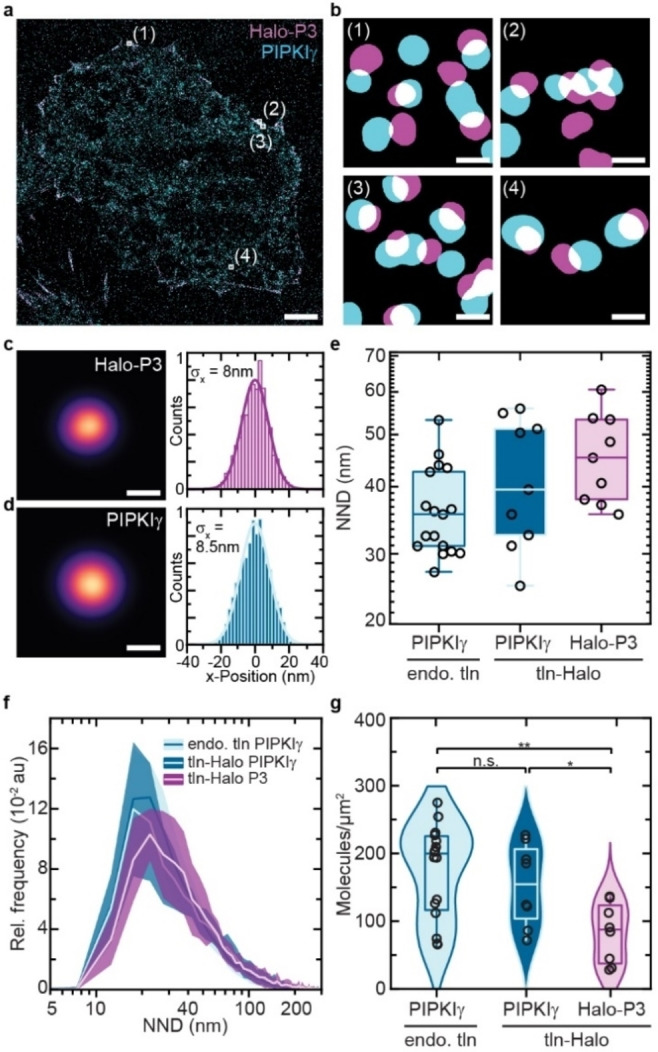
PIPKIγ‐PAINT enables high quality single‐molecule imaging matching DNA‐PAINT. **a**) Representative image of a talin‐1‐HaloTag expressing cell imaged with Cy3b‐PIPKIγ (blue) and DNA‐PAINT (Halo‐P3, magenta). **b**) Zoom‐ins highlighted in **a** reveal co‐localization of Cy3b‐PIPKIγ (blue) and DNA‐PAINT (magenta) signals. **c**) Aligned localization cloud from 100 individual talin‐1‐HaloTag localization clouds imaged with a P3 imager strand; cross‐sectional histogram with a Gaussian fit yielded a localization precision of 8 nm (n=100 localization clouds). **d**) Aligned talin localization cloud from 100 individual talin‐1‐HaloTag localization clouds obtained with PIPKIγ‐PAINT; cross‐sectional histogram with a Gaussian fit yielded a localization precision of 8.5 nm (n=100 localization clouds). **e**) Medians of nearest‐neighbor distances (NNDs) of endogenous talin in MEFs and talin‐1‐HaloTag in MKFs imaged with PIPKIγ‐PAINT and DNA‐PAINT (n_wt‐PIPKI_=20207; n_tln‐Halo‐PIPKI_=8215; n_tln‐Halo‐P3_=9077 talin NNDs; n_wt‐PIPKI_=17; n_tln‐Halo‐PIPKI_=9; n_tln‐Halo‐P3_=9 cells). **f**) PAINT‐based NND distributions (plotted as relative frequency (Rel. frequency) in arbitrary units (au)) in MEFs (light blue, PIPKIγ) and in talin‐1‐HaloTag expressing (*Tln1* ^−/−^ *Tln2* ^−/−^) MKFs (dark blue, PIPKIγ) indicate a shift toward smaller distances when compared with DNA‐PAINT measurements (magenta, Halo) (n_wt‐PIPKI_=20207; n_tln‐Halo‐PIPKI_=8215; n_tln‐Halo‐P3_=9077 talin NNDs; n_wt‐PIPKI_=17; n_tln‐Halo‐PIPKI_=9; n_tln‐Halo‐P3_=9 cells). **g**) Analyzing the molecular density of talin in MEFs (light blue, PIPKIγ) and talin‐1‐HaloTag expressing MKFs (dark blue, PIPKIγ) indicates less efficient labeling by DNA‐PAINT (magenta, Halo). Imaging endogenous talin in MEFs using Cy3b‐PIPKIγ yields a molecular density in FAs of about 200 molecules/μm^2^ (n_wt‐PIPKI_=17; n_tln‐Halo‐PIPKI_=9; n_tln‐Halo‐P3_=9 cells; p‐value_wt‐PIPKI, tln‐Halo‐PIPKI_=0.328; p‐value_tln‐Halo‐PIPKI, tln‐Halo‐P3_=0.01801; p‐value_wt‐PIPKI, tln‐Halo‐P3_=0.0033). Boxplots show median and 25^th^ and 75^th^ percentage with whiskers reaching to the last data point within 1.5×interquartile range. NND distributions show mean±standard deviations. Two‐sample t test: ** p≤0.01, * p≤0.05, n.s. (not significant) p>0.05. Scale bars: 2 μm (**a**), 40 nm (**b**), 18 nm (**c**, **d**).

To benchmark the PIPKIγ‐PAINT imaging with the established DNA‐PAINT methodology,^[16]^ we further examined the Exchange‐PAINT data from cells expressing the talin‐1‐HaloTag construct (Figure 3a, b). These analyses demonstrated that the Cy3b‐PIPKIγ data matched the HaloTag‐based DNA‐PAINT measurements. Both methods were characterized by similar localization precisions (DNA‐PAINT σ_x_=8 nm vs. Cy3b‐PIPKIγ σ_x_=8.5 nm; Figure 3c, d; Supporting Figures S4, 5), and Cy3b‐PIPKIγ indicated even slightly shorter nearest‐neighbor distances (NNDs) between individual talin‐1 localization clouds when compared to the DNA‐PAINT data sets. Calculating the molecular densities – using our previously established algorithm that correlates NNDs with molecular densities^[16]^ – revealed that the PIPKIγ data matched the value observed for endogenous talin in genetically unmodified fibroblasts, whereas the DNA‐PAINT approach indicated slightly lower molecular densities (Figure 3e–g; Supporting Figures S6, 7). Finally, we performed qPAINT experiments to confirm that indeed individual molecules are detected in our experiments. Consistent with our previous work,^[16]^ simultaneous recording of talin‐1 via DNA‐PAINT and DNA origami that harbor single‐binding sites demonstrated that individual talin molecules are imaged (Supporting Figure S8). Given the highly similar localization precision obtained by both methods, we conclude that PIPKIγ‐PAINT allows high‐quality, single‐molecule resolved talin measurements. As described for the IRIS approach,^[19]^ the method appears to benefit from high labeling efficiencies presumably because of the direct binding of Cy3b‐PIPKIγ to talin.

### Single‐molecule analyses of talin under physiologically relevant conditions using PIPKIγ‐PAINT

**Figure 4:**
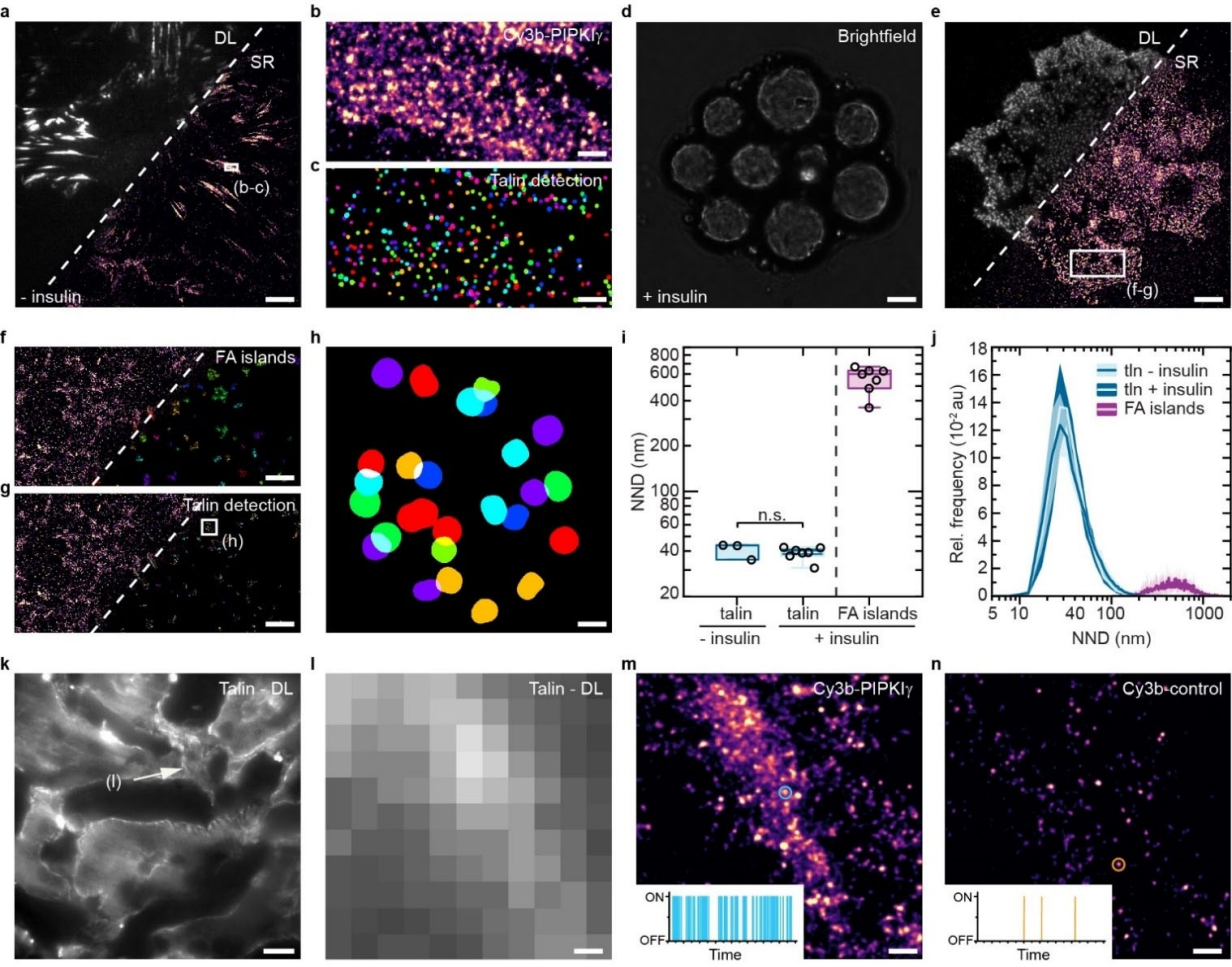
Molecular‐scale imaging of endogenous talin proteins in cells and tissues. **a**) Side‐by‐side view of a diffraction‐limited (DL) image of vinculin‐YPet and a super‐resolved (SR) image obtained with PIPKIγ‐PAINT of endogenous talin in mesenchymal stem cells. **b**–**c**) Zoom‐in of outlined area in **a** reveals distinct talin localization clouds in focal adhesions (FAs); a cluster detection algorithm indicates the individual talin molecules. **d**) Representative brightfield image of a mesenchymal stem cell differentiated by insulin treatment into an adipocyte; note the presence of lipid droplets indicating adipogenesis. **e**) Side‐by‐side view of a diffraction‐limited (DL) image of vinculin and the corresponding super‐resolved (SR) PAINT image of a Cy3b‐PIPKIγ‐labeled cell showing talin reorganization into punctate adhesion structures. **f**) Zoom‐in of outlined area in **e** reveals FA islands. **g**) Zoom‐in of outlined area in the Cy3b‐PIPKIγ image in **e** shows single talin molecules within the adhesion islands. **h**) Zoom‐in of outlined area in **g** showing single talin molecules within a distinct FA island. **i**) NND analysis of undifferentiated mesenchymal stem cells and adipocytes reveals that talin spacing (light blue) is unchanged despite an unusual large separation distance of individual FA islands (n_‐insulin_=18871; n_+insulin_=22855; n _FA islands_=1819 talin NNDs; n_‐insulin_=3; n_+insulin_=7; n _FA islands_=7 cells; p‐value=0.49). **j**) Distribution of the median from NND measurements (plotted as relative frequency (Rel. frequency) in arbitrary units (au)) of endogenous talin in mesenchymal stem cells and adipocytes upon insulin treatment; for comparison NND distributions of FA islands are shown (n_‐insulin_=18871; n_+insulin_=22855; n _FA islands_=1819 talin NNDs; n_‐insulin_=3; n_+insulin_=7; n _FA islands_=7 cells). **k**) Diffraction limited image of a kidney section from a homozygous talin‐1‐YPet mouse shows enrichment of talin‐1 at the basement membrane of renal tubules. **l**) Zoom‐in of marked area in **k. m**) PIPKIγ‐PAINT imaging reveals enrichment of talin molecules in the membrane of renal tubules. **n**) Exchange‐PAINT to the scrambled Cy3b‐control shows background binding events only. Boxplots show median and 25^th^ and 75^th^ percentage with whiskers reaching to the last data point within 1.5×interquartile range. NND distributions show mean±standard deviations. Two‐sample t test: n.s. (not significant) p>0.05. Scale bars: 10 μm (**k**), 7.5 μm (**a**), 7 μm (**d**, **e**), 1.4 μm (**f**, **g**), 250 nm (**b**, **c**), 150 nm (**l**–**n**), 50 nm (**h**).

Talin has been previously analyzed at single‐protein resolution in genetically modified cells[^14^, ^15^, ^16^] only, thus the available techniques are not applicable to a range of research questions. Therefore, we investigated whether PIPKIγ‐PAINT can be used to study physiologically relevant processes in unmodified cells and used mesenchymal stem cells (OP9 cells) as a model system.^[34]^ OP9 cells form prominent FAs in the undifferentiated state but dramatically change their adhesion structure during differentiation into adipocytes upon insulin treatment (Figure 4a–c). Indeed, PIPKIγ‐PAINT revealed distinct talin localization clouds in FAs of undifferentiated cells, with NNDs around 40 nm, similar to those observed in the fibroblasts shown above (Figure 4i). By contrast, the distribution of talin molecules was drastically altered upon differentiation (Figure 4d–g; Supporting Figure S9; Supporting Video 2, 3). While adhesion structures were hardly visible using conventional wide field microscopy and appeared as small dots, PIPKIγ‐PAINT showed that endogenous talin compartmentalized into distinct adhesion nanoclusters that were spaced about 600 nm apart, which is clearly distinct from the adhesion structures observed in thus far analyzed cell types.^[16]^ Intriguingly, the spacing between individual talin molecules, residing within these FA islands, did not change and remained at about 40 nm (Figure 4i‐j). Consistent with the observation that adipogenesis is accompanied by a drastic and functionally relevant change in integrin receptor expression,^[35]^ our data suggest a prominent role of these atypical cell‐matrix adhesions during differentiation. In addition, the experiments show that the combination of PIPKIγ labeling with PAINT can be utilized to investigate endogenously expressed talin without the requirement for genetic engineering to explore molecular processes under physiologically relevant conditions.

### PIPKIγ‐PAINT enables molecular‐scale analyses in mammalian tissues

Finally, we wanted to explore whether the Cy3b‐PIPKIγ probe can be applied to visualize the nanoscale organization of talin in tissues. Such experiments seemed especially relevant, because FAs are typically not observed in 3D environments and it is still controversially discussed how FA proteins assemble on molecular scales in vivo.[^17^, ^18^] To be able to thoroughly test this, we generated genetically modified mice, in which a YPet fluorophore was inserted into the talin‐1 gene, at a previously tested insertion site[^5^, ^6^, ^36^] corresponding to amino acid 447 (Supporting Figure S10). We reasoned that these animals would enable the visualization of talin‐1 in tissues with diffraction‐limited resolution, for example in the kidney,^[32]^ which would serve as a perfect control for testing the labeling specificity of Cy3b‐PIPKIγ. As expected, tissue sections from the kidney of adult, homozygous talin‐YPet mice showed a strong fluorescent signal in cell‐matrix interfaces (Figure 4k–l). PAINT imaging with Cy3b‐PIPKIγ of the same tissue section revealed distinct talin localization clouds specifically in these locations, with a NeNA‐based localization precision of about 9 nm (Figure 4m). As tissue samples are prone to unspecific binding of labeling probes, we included an additional specificity control by performing Exchange‐PAINT with the Cy3b‐control peptide. These experiments demonstrated isolated background signals only, which were again characterized by isolated, non‐repetitive binding events (Figure 4n). Hence, PIPKIγ‐PAINT enables visualizing endogenous talin proteins at molecular‐scale resolution even in tissue samples.

## Conclusion

In conclusion, we provide direct evidence that the previously reported PIPKIγ peptide[^19^, ^20^, ^23^] can be combined with PAINT imaging to allow the visualization and analysis of talin molecules in its native environment and at molecular scales. This further development of the IRIS technology now enables the analysis of talin in a wide range of biological systems including those that are inherently challenging or impossible to genetically engineer. Our experiments demonstrate that the quality of such measurements is comparable to state‐of‐the‐art single‐molecule localization technologies such as DNA‐PAINT[^16^, ^25^] obtaining localization precisions of <10 nm.

The first application of the PIPKIγ‐PAINT approach to mesenchymal stem cells uncovers a thus far undescribed adhesion structure characterized by nanoclusters with an atypical talin separation distance of 600 nm. How this particular mode of cell adhesion is mechanistically connected to stem cell differentiation needs further clarification, but the observation that mesenchymal stem cell differentiation is sensitive to cell‐substrate interactions^[37]^ and accompanied by a drastic change in integrin expression^[35]^ seems consistent with a functional role of the here identified talin structures.

Finally, we see significant potential in using the here described strategy to uncover how adhesion molecules such as talin assemble in vivo. It seems worth emphasizing that FAs are very prominent in cultured cells but hardly visible in 3D matrices or tissues. This inability to visualize and study FA proteins with sufficient resolution under those conditions has prevented a mechanistic, nanoscale understanding of cell‐matrix adhesion in tissues. The applicability of PIPKIγ‐PAINT to fixed tissue samples overcomes this obstacle and provides a way to clarify how cell‐matrix adhesions assemble in their natural environment. Potentially in combination with other technologies, allowing super‐resolution imaging in tissues,^[38]^ it should enable the molecular scale analysis of talin in numerous physiological and pathophysiological conditions such as cancer,^[39]^ fibrosis^[40]^ and aging.^[41]^ The ability of PIPKIγ to detect the mouse and the human talin protein will be especially useful in this context, as it should also enable the investigation of tissue samples and biopsies from patients.

Finally, we expect that the here used strategy to combine IRIS probes with PAINT imaging will further improve the applicability of single‐molecule localization microscopy to a wider range of research questions, especially those were the analysis of endogenous molecules in their natural context is critical.

## Experimental Section

A detailed experimental description is given in the Supporting Information.

## Conflict of interest

The authors declare no conflict of interest.

## Supporting information

As a service to our authors and readers, this journal provides supporting information supplied by the authors. Such materials are peer reviewed and may be re‐organized for online delivery, but are not copy‐edited or typeset. Technical support issues arising from supporting information (other than missing files) should be addressed to the authors.

Supporting Information

Supporting Information

Supporting Information

Supporting Information
