# Supplementary material for: Peptide‐PAINT Enables Investigation of Endogenous Talin with Molecular Scale Resolution in Cells and Tissues
Source: Chembiochem. 2021 Jul 30;22(19):2872–9. doi: 10.1002/cbic.202100301 (PMC8518977; doi:10.1002/cbic.202100301)
Supplement: Supplementary file 4 — Supporting Information [file CBIC-22-2872-s004.pdf]

ChemBioChem

Supporting Information

Peptide-PAINT Enables Investigation of Endogenous Talin with Molecular Scale Resolution in Cells and Tissues

Lisa S. Fischer,* Thomas Schlichthaerle, Anna Chrostek-Grashoff, and Carsten Grashoff*

Experimental Section	
Supplementary Figure 1	Individual signals used for average images and kinetic calculations of Cy3b-PIPKIγ data
Supplementary Figure 2	Labeling with the scrambled control peptide (Cy3b-control) yields unspecific binding events only
Supplementary Figure 3	Evaluation of Cy3b-PIPKIγ specificity using talin-deficient cells (*Tln*1$^{-/-}$*Tln*2$^{-/-}$)
Supplementary Figure 4	Localization precision of PIPKIγ-PAINT and DNA-PAINT
Supplementary Figure 5	Individual signals used for average images of PIPKIγ-PAINT and DNA-PAINT data in Figure 3
Supplementary Figure 6	Nearest-neighbor distances (NNDs) obtained from PIPKIγ-PAINT and DNA-PAINT measurements
Supplementary Figure 7	Detection of individual talin molecules using PIPKIγ-PAINT and a modified Ripley's K function analysis
Supplementary Figure 8	qPAINT evaluation confirms the detection of individual talin molecules per localization cloud
Supplementary Figure 9	PIPKIγ-PAINT of endogenous talin during mesenchymal stem cell differentiation into adipocytes
Supplementary Figure 10	Targeting strategy for the generation of Talin1-YPet mice
Supplementary Table 1	Cy3b-PIPKIγ and Cy3b-control amino acid sequence
Supplementary Table 2	Cy3b-PIPKIγ binding kinetics in PAINT nanoscopy
Supplementary Table 3	Ligands with conjugated DNA-PAINT docking sites and imager sequences
Supplementary Table 4	Parameters for DNA-PAINT and PAINT imaging using Cy3b-PIPKIγ
Supplementary Table 5	Sample size and experimental repeats
Supplementary References	

Experimental Section

Labeling probes, reagents and antibodies. Chloroalkane (CA; HaloTag ligand)-modified docking strands carrying an Atto488 dye at the 3'-end were custom-ordered from 'Biomers.net'. Imager strands with a Cy3b modification at the 3'-end were purchased from Eurofins; for oligonucleotide sequences see Supplementary Table 3. PIPKIγ-Peptide and Cy3b-control peptide both N-terminally modified with a Cy3b were synthesized by the Max Planck Institute of Biochemistry Peptide Synthesis Facility (Supplementary Table 1). For imaging, the following antioxidant stock solutions were used: 40x PCA solution (154 mg PCA diluted in 10 ml ddH_2O, pH 9.0), 100x Trolox (100 mg of Trolox in 430 µl Methanol, 345 µl NaOH (1M) and 3.2ml ddH_20) and 100x PCD solution (9.3 mg of PCD diluted in 13.3 ml of 50 % glycerol with 50 mM KCl, 1 mM EDTA and 100 mM Tris–HCl, pH 8.0). PCD, PCA and Trolox stocks were stored at -20 °C. In addition, the following antibodies and reagents were used: mouse anti-paxillin (BD Biosciences, 610052, dilution for immunofluorescence (IF): 1:200), mouse anti-vinculin (hVIN-1, Sigma, V9131; IF: 1:400), anti-mouse IgG Alexa Fluor-488 (Invitrogen, A11001; IF: 1:500), Triton X-100 (Roth, 3051.4), paraformaldehyde (Roth, 4980.1) and bovine serum albumin (Serva, 11930.03).

Plasmid construction. The talin expression constructs are based on human talin-1 cDNA (NM_006289) and human talin-2 cDNA (NM_015059). For internal tagging, a linker encoding for 5'SalI/3'NotI restriction sites was generated after the base pair encoding for aa 447 in talin-1 and aa 450 in talin-2, and HaloTag (Promega) was inserted by Gibson Cloning as described for talin-1 before.[1] The vinculin expression construct is based on human vinculin cDNA (NM_003373). The vinculin-YPet construct was generated by incorporating YPet internally after aa 883, as described earlier.[2] The assembled cDNAs were then transferred into a modified pLPCX (pLPCXmod)[3] and the correct sequence of all constructs was confirmed by DNA sequencing (Eurofins Genomics).

Generation and housing of Talin-1-YPet mice. A CRISPR-Cas9–mediated homology directed repair approach was used to insert the cDNA (amino acids 1-228) of YPet into exon 13 of the murine *Tln1* gene. The cDNA was inserted after the codon encoding for amino acid 447, as this site was previously used to tag talin-1 internally[3,4] which does not appear to affect talin function. A neomycin resistance cassette, flanked by loxP sites, was included in the repair template to enable selection of successfully targeted R1 ES cell clones (Supplementary Fig. 10). Modified ES cells were injected into C57BL/6N host blastocysts to obtain chimeric males, which were intercrossed with Cre-recombinase transgenic females (Tg(Nes-cre)1Wme)[5] to remove the neomycin selection cassette. The Cre transgene was subsequently bred out by crossing heterozygous males (talin-YPet/−, Cre) with C57BL/6N wild type females. Heterozygous (talin-YPet/+) animals were crossed to obtain homozygous (YPet/YPet) mice, which are phenotypically indistinguishable from wild type littermates. Mice were generated and housed under SPF barrier conditions at the animal facility of the Max Planck Institute of Biochemistry in Martinsried, Germany (room temperature: 22 ± 1.5 °C, relative humidity: 55 ± 5%, lighting: artificial with a light:dark cycle of 14:10 hours). All experiments involving animals were performed in accordance with animal welfare laws and approved by the Government of Upper Bavaria (55.2-1-54-2532-77-2015).

Cell culture. Murine kidney fibroblasts (MKFs) and murine embryonic fibroblasts (MEFs), were maintained in high glucose DMEM (ThermoFisher, 31966047) supplemented with 10 % Fetal Bovine Serum (ThermoFisher, 10270106) and 1 % Penicillin/Streptomycin (Sigma, P4333). The talin constructs were stably expressed by retroviral infection in MKFs, which are deficient for talin-1 and talin-2 ($Tln1^{-/-}Tln2^{-/-}$).[3] For imaging, 40,000 cells were seeded on ibidi µ-Dishes (ibidi, 81158). OP9 cells (ATCC® CRL-2749™) were cultured according to the manufacturer protocol. In brief, cells were maintained in Alpha Minimum Essential Medium without ribonucleotides and deoxyribonucleosides and completed with 2.2 g/L sodium bicarbonate and 20 % Fetal Bovine Serum. Differentiation of adipocytes was performed by replacing normal growth medium with insulin oleate (IO) medium and cells were fixed seven days after initiation of differentiation. IO contain MEM-α with 0.2% FBS, 175 nM insulin, 900 µM oleate bound to albumin (5.5:1 molar ratio), 100 U/ml penicillin, and 100 µg/ml streptomycin.[6]

Cell fixation and labeling of the HaloTag. Talin-1–HaloTag and Talin-2–HaloTag expressing cells were fixed with pre-warmed 4 % PFA solution for 10 min, washed 3× with phosphate-buffered solution (PBS) incubated for 90 min in 0.2 % Triton X-100/4 % BSA and stained with 1 µM of chloroalkane modified DNA docking strand in 4 % BSA solution overnight. Cells were then washed 3× for 5 min in 1× PBS, incubated with a 1:3 dilution of 90 nm gold particles (Cytodiagnostics, G-90-100) as drift markers in 1× PBS for 5 min, washed again 3× 5 min in PBS, and immediately imaged.

Cell fixation and labeling of wildtype cells with antibody co-staining. Cells were fixed with pre-warmed 4 % PFA solution for 10 min and washed 3× with PBS. Subsequent incubation with 1 mg/ml Sodium-Borohydride for 7 min at room temperature and followed by 3× washing steps with 1× PBS. Next, the sample was incubated for 90 min in 0.2 % Triton X-100/4 % BSA and stained with a paxillin (1:200) or a vinculin antibody (1:400) in 4 % BSA solution overnight. Cells were washed 3× for 5 min in 1× PBS and incubated with the secondary antibody (1:500) in 4 % BSA solution for 1 h at room temperature. Then, cells were washed 3× for 5 min in 1× PBS and incubated with a 1:3 dilution of 90 nm gold particles (Cytodiagnostics, G-90-100) as drift markers in 1× PBS for 5 min, washed again 3× 5 min in PBS, and immediately imaged.

Tissue fixation and sample preparation. To perform PIPKIγ-PAINT imaging in tissue, 5 µm sections were fixed with pre-warmed 4 % PFA solution for 10 min, washed 3× with PBS and incubated for 90 min in 0.2 % Triton X-100/4 % BSA. The tissue section was washed 3× for 5 min in 1× PBS and incubated with a 1:3 dilution of 90 nm gold particles (Cytodiagnostics, G-90-100) as drift markers in 1× PBS for 5 min, washed again 3× 5 min in PBS, and immediately imaged.

DNA origami assembly. DNA origami was prepared as described previously.[1,7,8] In brief, DNA origami self-assembly was accomplished via a thermal ramp using a thermocycler in a reaction mix including 10nM p7249 scaffold strand M13mp18 (tilibit nanosystems), 100nM folding staples (Eurofins), 1 µM P3 docking strand (Eurofins), and 10 nM biotinylated staples (Eurofins) in 1x TE buffer (12.5 mM $MgCl_2$).[8] Subsequent, the origami was purified using Amicon Ultra Filter (50K, 0.5 ml; Merck Millipore) and stored in folding buffer (10 mM $MgCl_2$, 5 mM Tris, 1 mM EDTA) at -20 °C.

qPAINT experiment with DNA origami. qPAINT experiments were conducted as described before.[1,7,8] Cells were seeded, fixed, permeabilized, and labeled with P3 docking strand. Before imaging, cells were washed 3× times with 1× PBS and incubated for 10 min with 200 µl BSA-Biotin solution (1 mg/ml BSA-Biotin in buffer C (1× PBS + 500 mM NaCl)). The dish was then carefully washed with buffer C, 200 µl streptavidin solution (0.5 mg/ml in buffer C) was added, and incubated for another 10 min. Next, the dish was washed 3× times with buffer C and incubated with 200 µl of biotin-labeled DNA origami solution (200 pM in buffer C) for 60 min. Afterwards, DNA origami solution was removed, washed carefully with buffer C and imaging buffer was added.

Super-resolution microscopy. Fluorescence imaging was carried out on a commercial Nikon Ti-E N-SIM/N-STORM setup equipped with the Perfect Focus System. The objective was a CFI SR APO TIRF 100xH oil, NA 1.49, WD 0.12 mm (Nikon) using immersion oil from Nikon (nd: 1.515). The TIRF angle was adjusted for highest signal-to-noise ratio prior to imaging. The light source was controlled by a LU-NV Laser Unit with a 488 nm (max. 70mW at sample) and a 561 nm (max. 70mW at sample) laser line using independent TIRF filter cubes (Chroma filter cube Nikon TIRF 488: 525/50 and Nikon TIRF 561: 605/50). Images were collected with a sCMOS camera (Andor Zyla 5.5) without further magnification resulting – after 2x2 binning – in an effective pixel size of 130 nm per pixel. The system was controlled by the NIS-Elements (Nikon) software.

DNA-PAINT and PIPKIγ-PAINT imaging. FAs of cells or tissue sections were brought to focus using a 488 nm excitation. For DNA-PAINT and PIPKIγ-PAINT imaging, samples were imaged with 561 nm excitation. Imager strand concentration for DNA-PAINT was set to 2.5 nM; Cy3b-PIPKIγ and the Cy3b-control peptide were used at a concentration between 7–30 nM depending on the cell lines, tissue section, and experimental setup. Imaging was performed in the presence of an oxygen scavenging and triplet state quencher system consisting of a solution of 1x PCA (Stock 40x PCA solution; Sigma, 37580), 1x PCD (Stock 100x PCD solution; Sigma, P8279) and 1x Trolox (Stock 100x Trolox solution; Sigma, 238813) in 1× PBS + 500 mM NaCl. Typically, 80,000 frames at 100 ms exposure time were acquired for DNA-PAINT imaging, and 100,000–140,000 frames at 40 ms for PIPKIγ -PAINT measurements. Super-resolution tissue imaging was performed over 140,000 frames and 40 ms integration time using Cy3b-PIPKIγ or Cy3b-control peptide. Exact parameter sets are summarized in Supplementary Table 4.

Multiplexed imaging with Exchange-PAINT. In the first step, Cy3b-PIPKIγ was imaged using a concentration of 15 nM for MKFs (*Tln1*$^{-/-}$*Tln2*$^{-/-}$) expressing talin-1–HaloTag or talin-2–HaloTag; 30 nM was used for imaging tissue sections of talin-1-YPet mice. After washing, 2.5 nM Cy3b-P3 was added to image talin-1–HaloTag or talin-2–HaloTag in MKFs, and 30 nM Cy3b-control peptide was added for tissue imaging in kidney sections.

Image reconstruction. Images were reconstructed with the Picasso Software (available on https://github.com/jungmannlab/picasso). Drift correction was performed stepwise starting with the gold nanoparticles for global drift correction followed by image sub-stack cross-correlation analysis. Localization precision was determined by NeNA-based analysis.[9] Cy3b-PIPKIγ kinetic was analyzed using the information of individual talin localization clusters via the 'pick' tool of the Picasso software and kinetic information was isolated using the 'save pick properties' command. In addition, data were plotted and further analyzed using Origin 2020b.

Data processing and analysis. Cluster analysis was performed with a modified Ripley's K function by calculating the amount of neighboring localizations within a radius r of 10 nm – defined by the NeNA localization precision – for each localization.[10] Then, a gradient ascend was used to identify the cluster center exhibiting the highest number of neighbors within the radius. All localizations within the radius of the cluster center were then assigned to the cluster. Next, detected clusters were filtered by a threshold number of localizations, for repetitive visits via a mean-frame analysis of occurring localizations within 20–80 % of the total acquisition time

and by a frame segmentation filter (i.e. a window filter which detects repetitive visits over the whole course of imaging and removes clusters based on unspecific sticking). Cluster center positions were then used for subsequent nearest neighbor calculations. For NND calculations, we used k-d tree analysis[11] to calculate the nearest neighbor within the dataset. Molecular densities were calculated by dividing the determined number of localization clouds within the FA mask by the respective FA mask area.

To detect adhesion islands in adipocytes, we used DBSCAN (density-based clustering of application with noise) as a data clustering algorithm.[8,12] DBSCAN detects adhesion islands by searching for minimal numbers of localizations within a circle of the radius ε; depending on the size of the adhesion islands that range between 0.7 – 0.9 pixel. The algorithm utilizes a minimum number of points (MinPts set to 300 – 500 localizations per adhesion island) within an area of the circle as a second parameter. ε was set depending on the size of the adhesion islands and MinPts on the amount of talin molecules and thus localizations residing in an adhesion island.

Averaging of single sites. Talin sites were manually selected or with the 'Pick similar' option of the Picasso software, aligned by their center of mass and averaged using the Picasso average tool to measure the size and heterogeneity of individual clusters.

qPAINT analysis. The reconstructed, undrifted cell image was analyzed as described before.[1,8] In brief, the Picasso Render module was used to selected 600 single origami binding sites with a constant pick radius using the "pick" tool. Next, the isolated binding sites were calibrated to one unit per binding site and the influx rate per single binding site was determined. Afterwards, 600 talin localization clouds were selected using the same pick radius and the mean number of binding sites per selected localization cloud were determined based on the calibrated influx rate.

Statistical analysis. To determine statistical significance, two-sample t-test were used with a p-value less than 0.05 indicating a statistically significant difference (n.s. $P > 0.05$; * $P \leq 0.05$; ** $P \leq 0.01$; *** $P \leq 0.001$). Normality of the data was tested using the Shapiro-Wilk test. Sample size and repeats are summarized in Supplementary Table 5.

Supplementary Figures

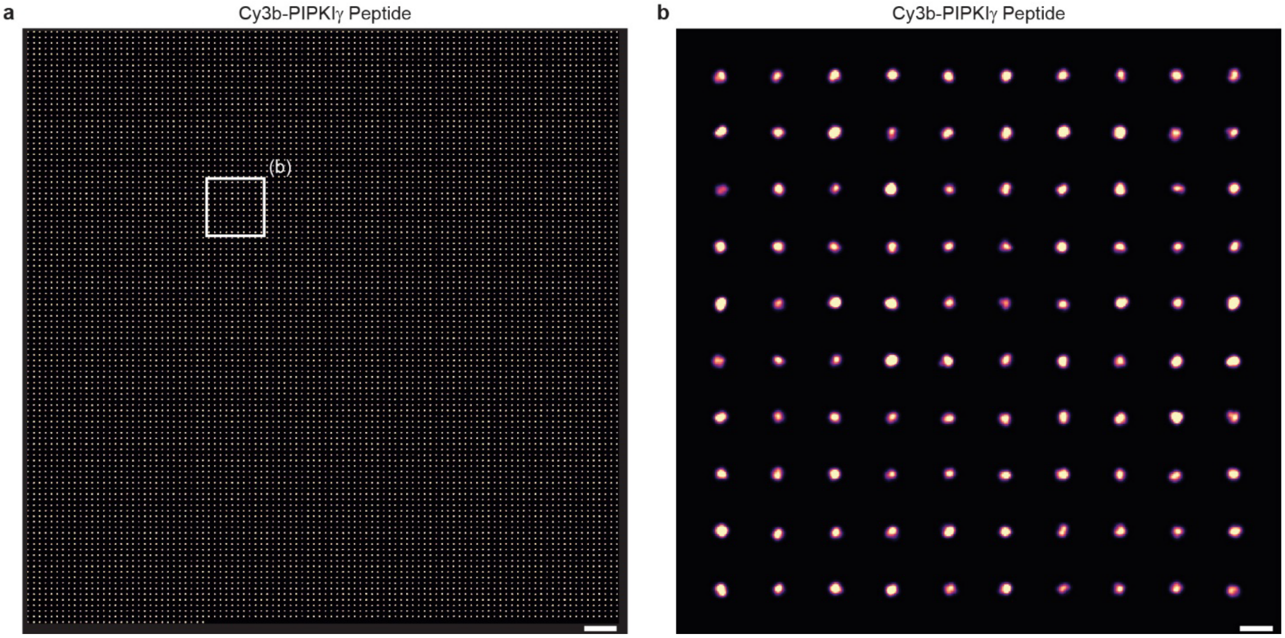

Supplementary Figure 1 | Individual signals used for average images and kinetic calculations of Cy3b-PIPKIγ data. a, 10,843 individual Cy3b-PIPKIγ localization clouds were used for calculating the binding kinetics to verify the peptide as a probe for PAINT imaging (n = 10,832 localization clouds). **b,** Zoom-in (highlighted in **a**) reveals single localization signals acquired with Cy3b-PIPKIγ. Scale bars: 1.6 μm (**a**), 150 nm (**b**).

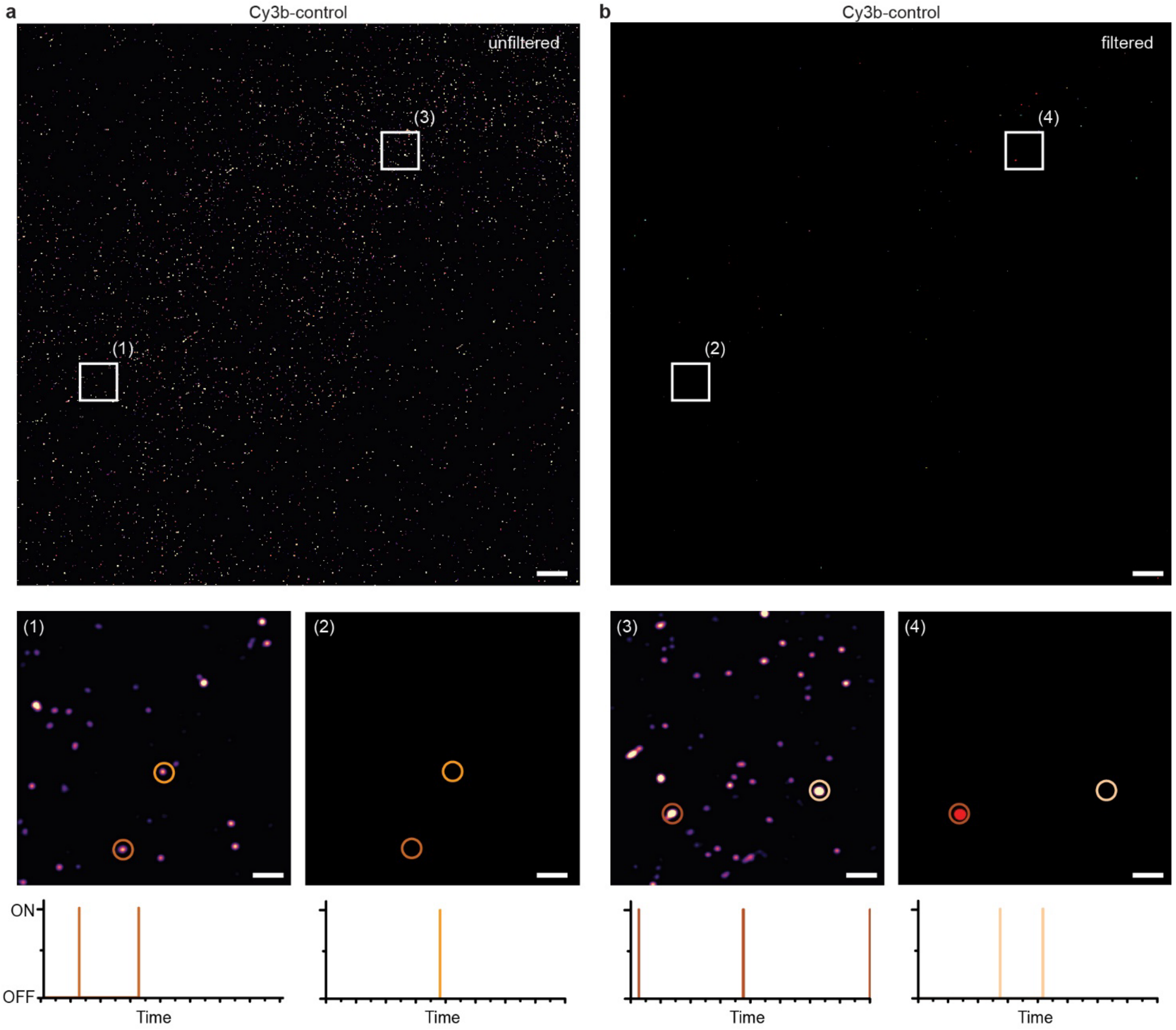

Supplementary Figure 2 | Labeling with the scrambled control peptide (Cy3b-control) yields unspecific binding events only. **a,** Zoom into focal adhesion area of a wildtype murine embryonic fibroblast (MEF) reveals disperse clusters using Cy3b-control. (1) and (3) are zoom-ins highlighted in **a** displaying the binding frequencies characteristic of unspecific binding events. **b,** Corresponding area after filtering and cluster detection. (2) and (4) are zoom-ins highlighted in **b** and correspond to the images in (1) and (3) after filtering. Traces below display the original binding frequencies indicating unspecific labeling. Scale bars: 1 µm (**a, b**), 130 nm ((1) – (4)).

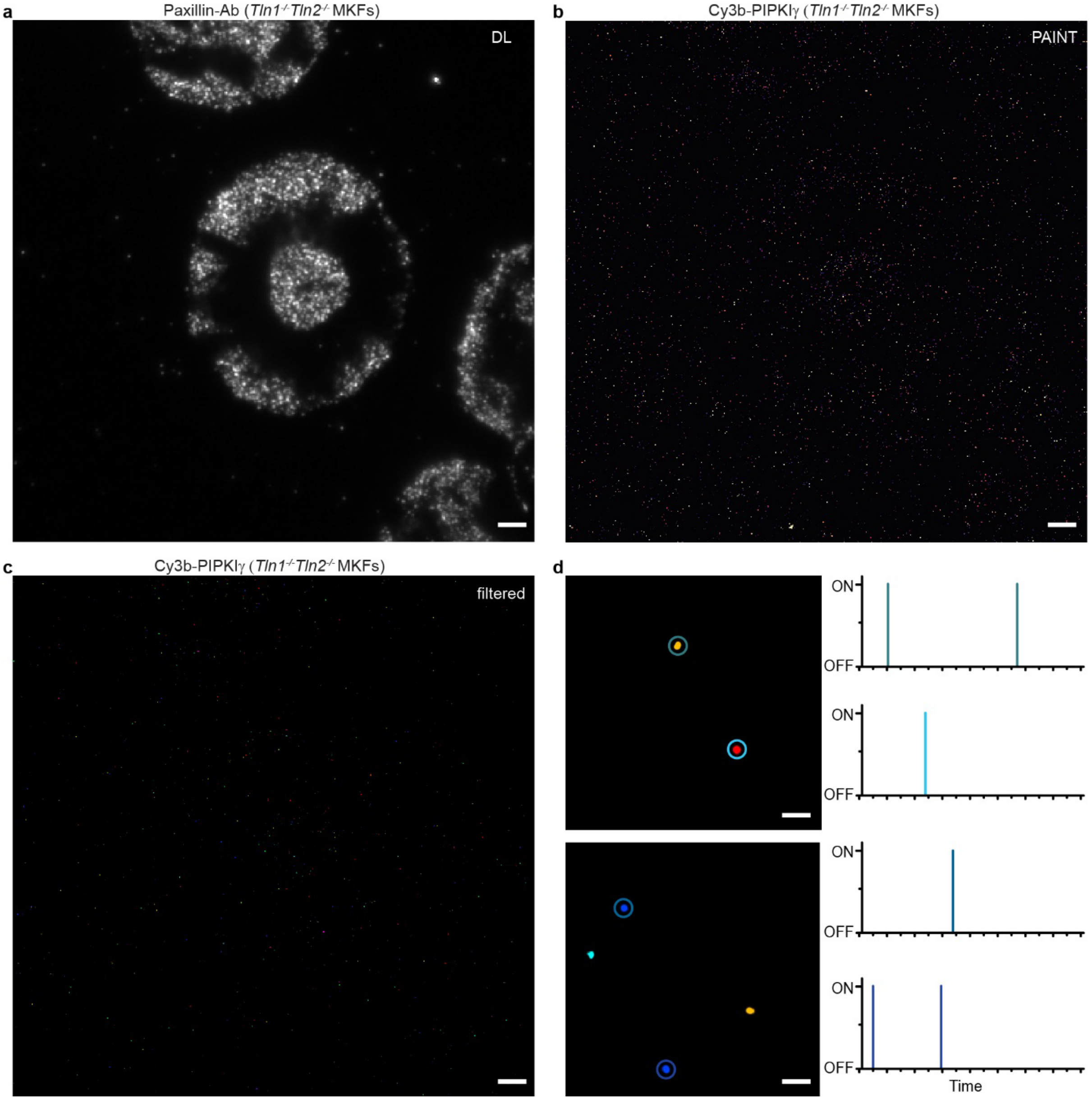

Supplementary Figure 3 | Evaluation of Cy3b-PIPKIγ specificity using talin-deficient cells (*Tln1*$^{-/-}$*Tln2*$^{-/-}$). a, Diffraction-limited (DL) image of *Tln1*$^{-/-}$*Tln2*$^{-/-}$ cells stained with the FA marker paxillin. **b,** Corresponding PAINT image acquired with Cy3b-PIPKIγ showing only isolated localization signals. **c,** Image processing using a Ripley's K function algorithm removes unspecific signals. **d,** Zoom-ins and display of the binding frequencies of localization signals imaged with Cy3b-PIPKIγ showing few remaining background signals (sticking events). Scale bars: 2.5 µm (**a**-**c**), 130 nm (**d**).

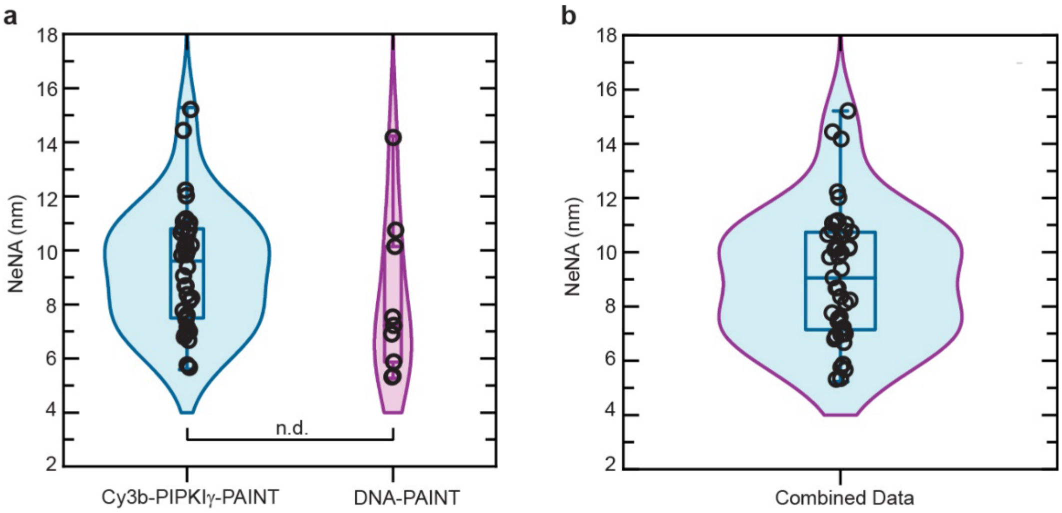

Supplementary Figure 4 | Localization precision of PIPKIγ-PAINT and DNA-PAINT. a, Nearest neighbor-based analysis (NeNA) of Cy3b-PIPKIγ-PAINT data (blue) reveals a localization precision of 9.6 nm. Localization precision in DNA-PAINT measurements (magenta) is highly similar ($n_{Cy3b\text{-}PIPKI\gamma} = 40$; $n_{Halo\text{-}P3} = 9$; p-value = 0.18204). **b,** NeNA-based calculation of all PAINT measurements indicates an average localization precision of 9 nm ($n_{Cy3b\text{-}PIPKI\gamma} = 49$). Boxplots show median and 25th and 75th percentage with whiskers reaching to the last data point within 1.5x interquartile range.

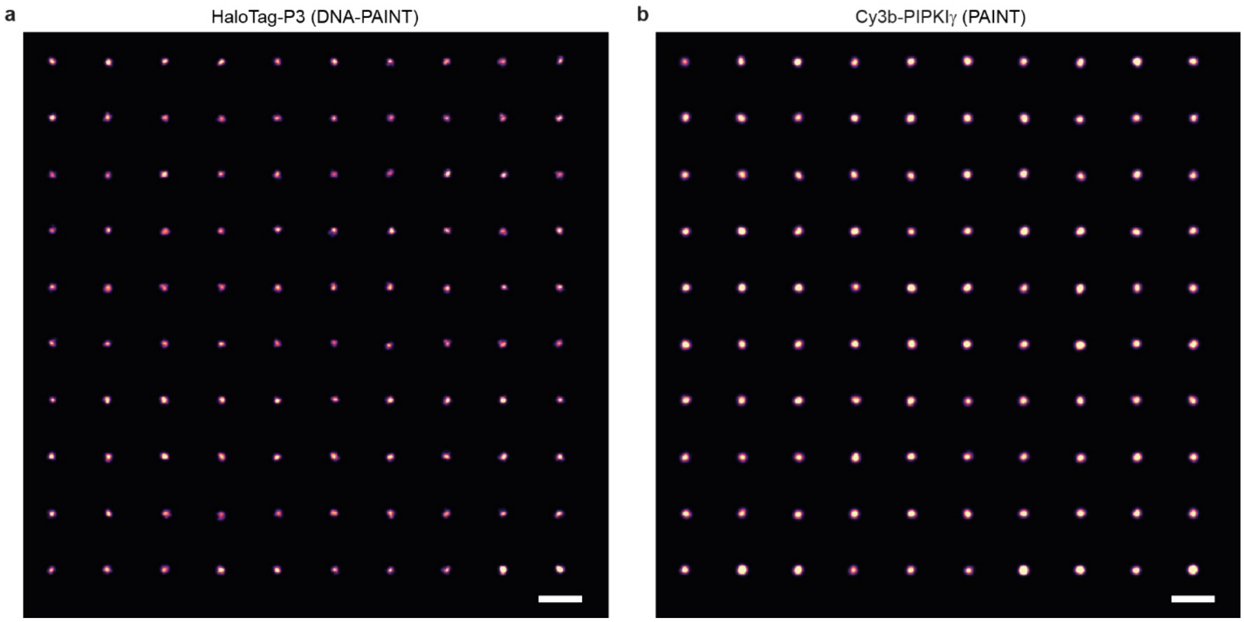

Supplementary Figure 5 | Individual signals used for average images of PIPKIγ-PAINT and DNA-PAINT data in Figure 3. a, 100 picked signals from individual talin-1 localization clouds acquired from *Tln1*$^{-/-}$*Tln2*$^{-/-}$ murine kidney fibroblasts (MKFs) reconstituted with talin-1–HaloTag with DNA-PAINT; these data were used for generating the aligned, average image and the cross-sectional histogram in Fig. 3c (n = 100 localization clouds). **b,** 100 picked signals from individual talin-1 localization clouds acquired from *Tln1*$^{-/-}$*Tln2*$^{-/-}$ MKFs reconstituted with talin-1–HaloTag by PIPKIγ-PAINT; these data were used for generating the aligned, average image and the cross-sectional histogram shown in Fig. 3d (n = 100 localization clouds). Scale bars: 200 nm (**a, b**).

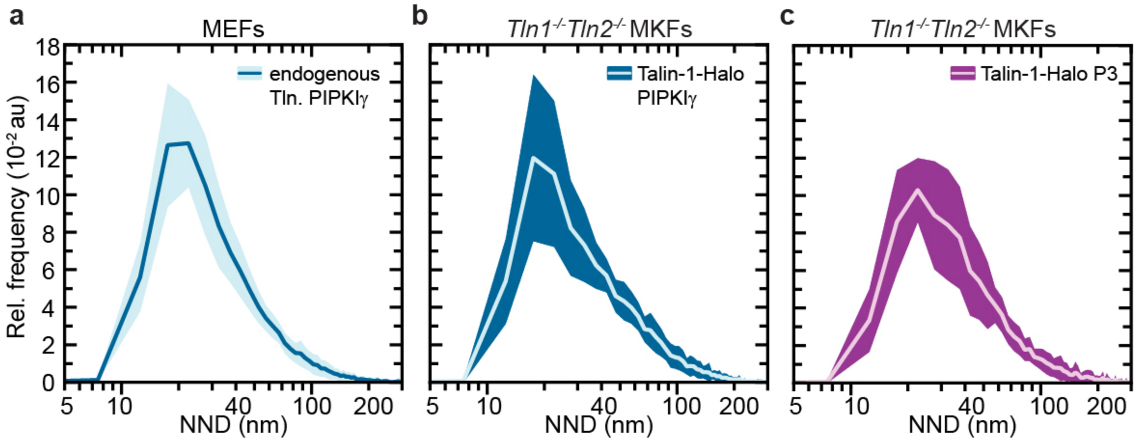

Supplementary Figure 6 | Nearest-neighbor distances (NNDs) obtained from PIPKIγ-PAINT and DNA-PAINT measurements. **a,** NND distribution of endogenous talin in wildtype murine embryonic fibroblasts (MEFs; dark blue line); data were acquired using Cy3b-PIPKIγ (n = 20,207 talin NNDs; n = 17). **b,** NND distribution of talin-1 in *Tln1*$^{-/-}$*Tln2*$^{-/-}$ murine kidney fibroblasts (MKFs) reconstituted with talin-1–HaloTag (light blue line); data were acquired using PIPKIγ-PAINT (n = 8,215; n = 9). **c,** NND distribution of talin-1 in *Tln1*$^{-/-}$*Tln2*$^{-/-}$ MKFs reconstituted with talin-1-HaloTag; images were acquired by DNA-PAINT using a P3 imager strand (magenta) (n = 9,077; n = 9 cells). NND distribution are plotted as relative frequency (Rel. frequency) in arbitrary units (au) and indicate the mean ± std (shaded area).

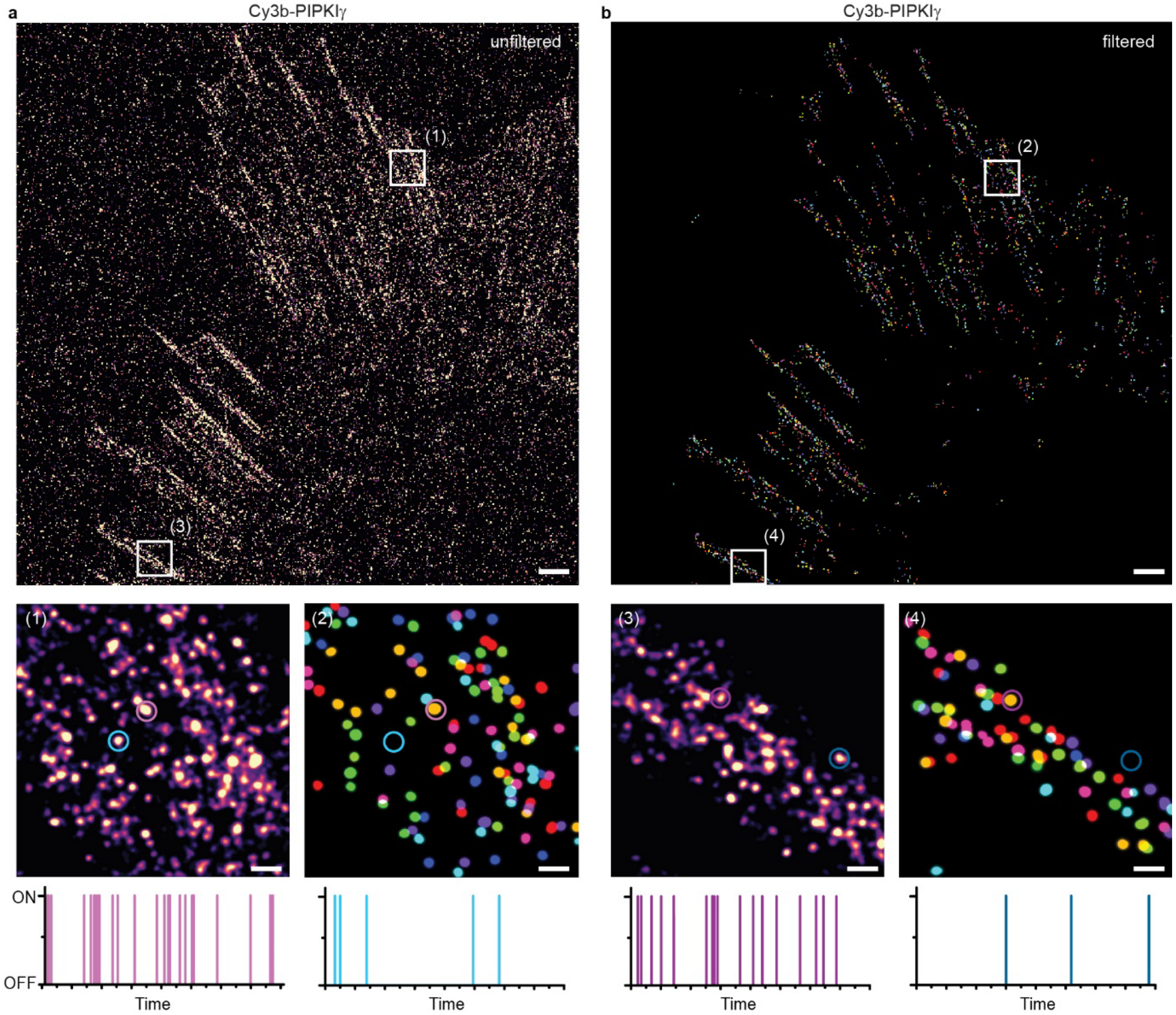

Supplementary Figure 7 | Detection of individual talin molecules using PIPKIγ-PAINT and a modified Ripley's K function analysis. a, Zoom into focal adhesion area of a Cy3b-PIPKIγ labeled wildtype MEF reveals individual talin molecules. (1) and (3) show zoom-ins highlighted in **a** displaying the binding frequencies of repetitively visited localization clouds (magenta colors, bottom left) acquired with Cy3b-PIPKIγ. **b,** Corresponding area after filtering and cluster detection. (2) and (4) are zoom-ins highlighted in **b** and corresponding images of (1) and (3). Traces below display the binding frequencies of removed clusters by automated filtering steps (blue colors, bottom right) acquired with Cy3b-PIPKIγ. Scale bars: 1 µm (**a, b**), 130 nm ((1) – (4)).

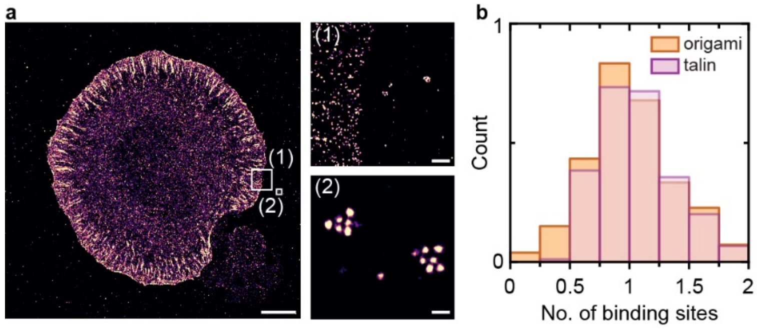

Supplementary Figure 8 | qPAINT evaluation confirms the detection of individual talin molecules per localization cloud. a, DNA origami structures were placed next to a talin-1-HaloTag expressing cell for the calculation of molecules per localization cloud. (1) shows DNA origami at the cell edge. (2) Zoom onto DNA origami structures. **b,** Histogram showing the absolute number of binding sites on DNA origami and talin localization clouds labeled with a P3 imager strand, indicating the detection of single binding sites. Scale bar: 7 µm (**a**), 300 nm (**a** (1, 2)).

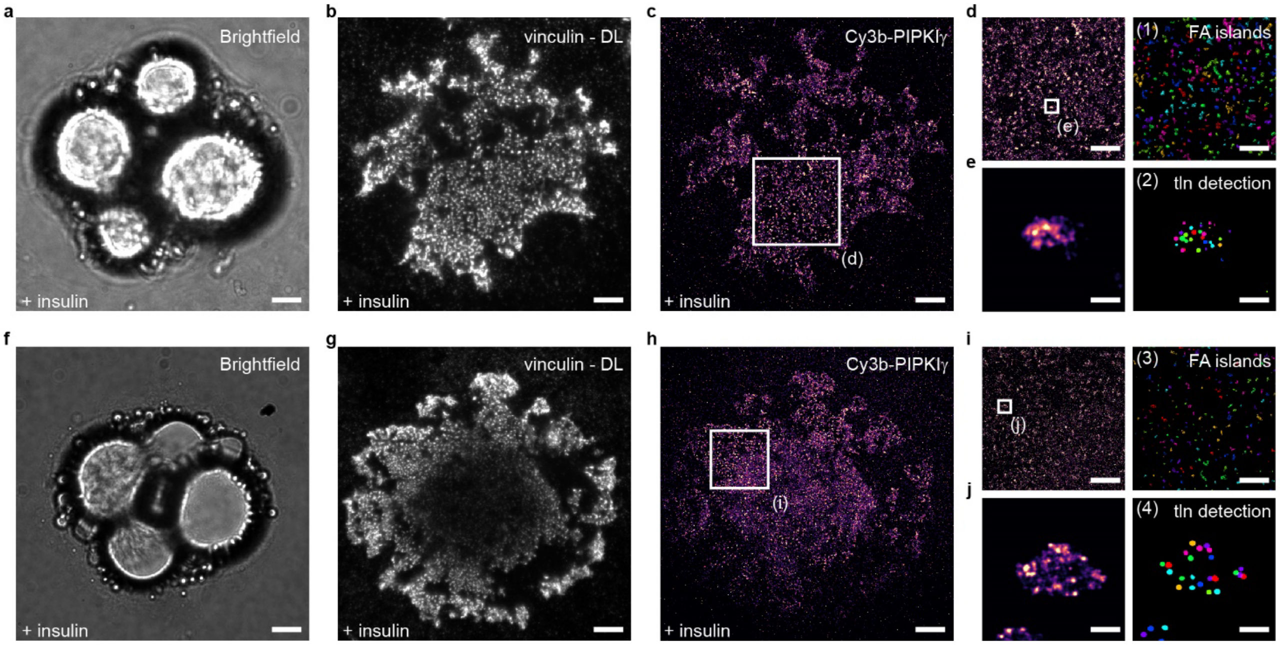

Supplementary Figure 9 | PIPKIγ-PAINT of endogenous talin during mesenchymal stem cell differentiation into adipocytes. a, f, Representative brightfield images of mesenchymal stem cells differentiated into adipocytes by insulin stimulation; note the presence of lipid droplets indicating adipogenesis. **b, g,** Diffraction-limited image of vinculin in adipocytes indicates punctate adhesion structures. **c, h,** Corresponding super-resolved PIPKIγ-PAINT images of adipocytes. **d,** Zoom-in of outlined area in the PAINT image in **c** reveals drastically altered adhesion morphology. Cell adhesions appear as focal adhesion (FA) islands as detected by DBSCAN-based image analysis (1). **e,** Zoom-in of outlined area in **d** shows individual talin molecules residing in distinct FA islands (2). **i,** Zoom-in of outlined area in the Cy3b-PIPKIγ-PAINT image in **h** reveals focal adhesion (FA) islands (3). **j,** Zoom-in of outlined area in **i** shows individual talin molecules residing in distinct FA islands. Scale bars: 7.5 μm (**f-h**), 5 μm (**a-c**), 3 μm (**d, i**), 220 nm (**e**), 200 nm (**j**).

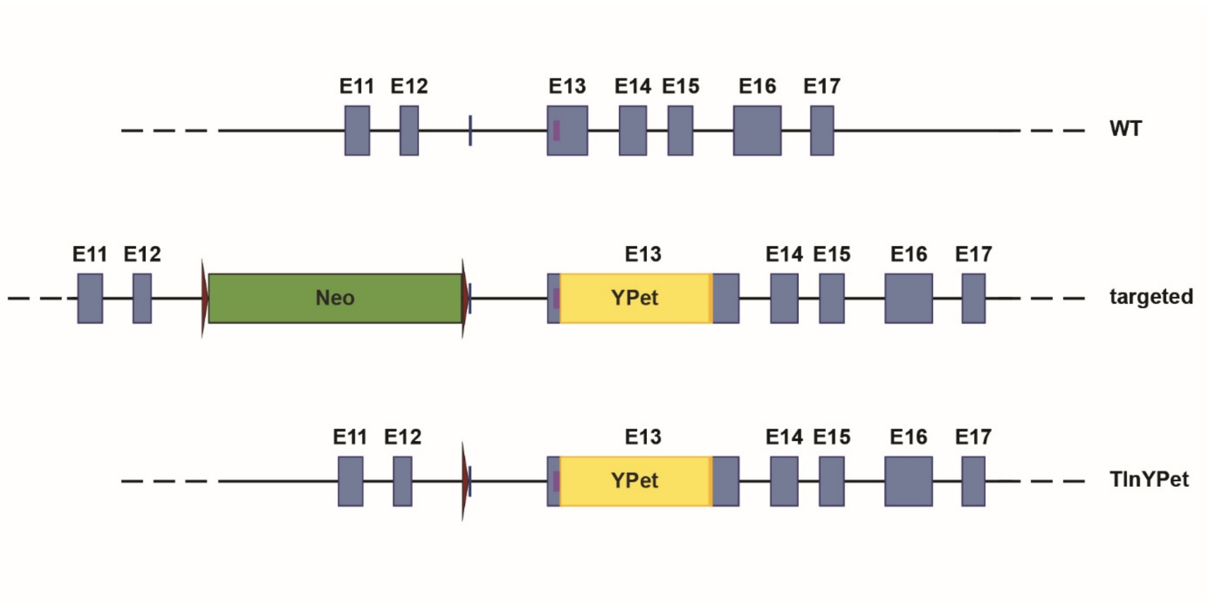

Supplementary Figure 10 | Targeting strategy for the generation of Talin1-YPet mice. Schematic representation of the CRISPR-Cas9–mediated targeting strategy to integrate a YPet fluorophore cDNA into exon 13 (E13) of the murine *Tln1* gene (WT). A neomycin-resistance cassette (Neo), flanked by loxP sites (triangles), was included in the repair template to facilitate selection of modified ES cells, carrying the targeted allele (targeted), which were used to generate chimeric animals. Subsequent breeding with Cre transgenic mice led to excision of the Neo cassette, resulting in the modified talin-1-YPet allele. A sgRNA target site in E13, here indicated with a purple rectangle, was used.

Supplementary Tables

Supplementary Table 1 | Cy3b-PIPKIγ and Cy3b-control amino acid sequence.

Name	Sequence (aa)	N-term	Peptide length	Molecular weight (g/mol)
Cy3b-PIPKIγ	PTDERSWVYSPLHYSAQAPPASDGESDT	Cy3b	28	3 606.54
Cy3b-control	PSDDPSQVPGLSADTAESRTYAPWHESY	Cy3b	28	3 606.5

Supplementary Table 2 | Cy3b-PIPKIγ binding kinetics in PAINT nanoscopy.

**Name**	**τ_b (ms)**	**τ_b (ms) -std**	**τ_d (s)**	**τ_d (s) - stdv**	**picked clusters**
Cy3b-PIPKIγ	222.91	98.76	167.57	74.84	10,843

Supplementary Table 3 | Ligands with conjugated DNA-PAINT docking sites and imager sequences.

**Name**	**Sequence**	**5'-mod**	**3'-mod**	**Company**
CA-P3	TTT CTT CAT TAT T	Chloroalkane	Atto-488	Biomers
P3-Imager (8er)	AAT GAA GA	None	Cy3b	Eurofins

Supplementary Table 4 | Parameters for DNA-PAINT and PAINT imaging using Cy3b-PIPKIγ.

Cell Type	Imaging Probe	Concentration	Number of Frames	Exposure time
MKFs (*Tln1*$^{-/-}$*Tln2*$^{-/-}$)	P3 imager strand	2.5 nM	80 000	100 ms
MKFs (*Tln1*$^{-/-}$*Tln2*$^{-/-}$)	Cy3b-PIPKIγ	15 nM	100 000	40 ms
MEFs	Cy3b-PIPKIγ	7 nM	100 000	40 ms
MEFs	Cy3b-control	7 nM	100 000	40 ms
OP9 cells	Cy3b-PIPKIγ	20 nM	140 000	40 ms
Tissue section	Cy3b-PIPKIγ	30 nM	140 000	40 ms
Tissue section	Cy3b-control	30 nM	140 000	40 ms

Supplementary Table 5 | Sample size and experimental repeats.

Figure Notation	Batch Name	Experimental Days (N)	Sample Number (n)
Figure 1e, f, i, j	Peptide kinetic	1	10 843 clusters
Figure 1g	NeNA	7	40
Figure 3c	DNA-PAINT Resolution	1	100 aligned clusters
Figure 3d	Cy3b-PIPKIγ-PAINT Resolution	1	100 aligned clusters
Figure 3e	NND comparison wild type	3	17 cells
Figure 3e	NND comparison Fibroblasts	3	35 cells (17, 9, 9)
Figure 3f	NND distributions	6	35 cells (17, 9, 9)
Figure 3g	Molecular Densities	6	35 cells (17, 9, 9)
Figure 4i	NND comparison Stem cells	2, 1	10 cells (7, 3)
Figure 4j	NND Distributions	2, 1	10 cells (7, 3)
Supplementary Fig. 1	Picks used for average	1	10,843 clusters
Supplementary Fig. 4	NeNA	6	35 cells (27, 8)
Supplementary Fig. 5a	Picks used for average	1	100 localization clouds
Supplementary Fig. 5b	Picks used for average	1	100 localization clouds

Supplementary Fig. 6a	Talin distribution in MEFs	3	17 cells
Supplementary Fig. 6b, c	Talin distribution in MKFs	3	18 cells (9 cells with Exchange-PAINT)
Supplementary Fig. 8b	qPAINT experiment	1	600 talin & 600 origami localization clouds

Supplementary References

[1] L. S. Fischer, C. Klingner, T. Schlichthaerle, M. T. Strauss, R. Böttcher, R. Fässler, R. Jungmann, C. Grashoff, *Nature communications* **2021**, *12*, 919.

[2] V. Kanoldt, C. Kluger, C. Barz, A.-L. Schweizer, D. Ramanujam, L. Windgasse, S. Engelhardt, A. Chrostek-Grashoff, C. Grashoff, *Nature communications* **2020**, *11*, 6403.

[3] K. Austen, P. Ringer, A. Mehlich, A. Chrostek-Grashoff, C. Kluger, C. Klingner, B. Sabass, R. Zent, M. Rief, C. Grashoff, *Nat Cell Biol* **2015**, *17*, 1597.

[4] P. Ringer, A. Weißl, A.-L. Cost, A. Freikamp, B. Sabass, A. Mehlich, M. Tramier, M. Rief, C. Grashoff, *Nature Methods* **2017**, *14*, 1090.

[5] U. A. Betz, C. A. Vosshenrich, K. Rajewsky, W. Müller, *Current biology : CB* **1996**, *6*, 1307.

[6] a) N. E. Wolins, B. K. Quaynor, J. R. Skinner, M. J. Schoenfish, A. Tzekov, P. E. Bickel, *The Journal of biological chemistry* **2005**, *280*, 19146; b) N. E. Wolins, B. K. Quaynor, J. R. Skinner, A. Tzekov, C. Park, K. Choi, P. E. Bickel, *Journal of lipid research* **2006**, *47*, 450.

[7] K. F. Wagenbauer, F. A. S. Engelhardt, E. Stahl, V. K. Hechtl, P. Stömmer, F. Seebacher, L. Meregalli, P. Ketterer, T. Gerling, H. Dietz, *ChemBioChem* **2017**, *18*, 1873.

[8] J. Schnitzbauer, M. T. Strauss, T. Schlichthaerle, F. Schueder, R. Jungmann, *Nature Protocols* **2017**, *12*, 1198.

[9] U. Endesfelder, S. Malkusch, F. Fricke, M. Heilemann, *Histochemistry and cell biology* **2014**, *141*, 629.

[10] J. Hellmeier, R. Platzer, A. S. Eklund, T. Schlichthaerle, A. Karner, V. Motsch, M. C. Schneider, E. Kurz, V. Bamieh, M. Brameshuber et al., *Proceedings of the National Academy of Sciences of the United States of America* **2021**, *118*.

[11] Fabian Pedregosa, Gaël Varoquaux, Alexandre Gramfort, Vincent Michel, Bertrand Thirion, Olivier Grisel, Mathieu Blondel, Peter Prettenhofer, Ron Weiss, Vincent Dubourg et al., *Journal of Machine Learning Research* **2011**, *12*, 2825.

[12] M. Ester, H.-P. Kriegel, J. Sander, X. Xu in *KDD'96*, AAAI Press, **1996**, pp. 226–231.
